# Bio-Mediated Synthesis of Reduced Graphene Oxide Nanoparticles from *Chenopodium album*: Their Antimicrobial and Anticancer Activities

**DOI:** 10.3390/nano10061096

**Published:** 2020-06-01

**Authors:** Mohammad Faisal Umar, Faizan Ahmad, Haris Saeed, Saad Ali Usmani, Mohammad Owais, Mohd Rafatullah

**Affiliations:** 1 School of Industrial Technology, Universiti Sains Malaysia, Penang 11800, Malaysia; faisalumar@student.usm.my; 2 Department of Post Harvest Engineering and Technology, Faculty of Agricultural Sciences, Aligarh Muslim University, Aligarh 202002, India; faizan.phe@amu.ac.in (F.A.); sausmani@myamu.ac.in (S.A.U.); 3 Molecular Immunology Lab, Interdisciplinary Biotechnology Unit, Aligarh Muslim University, Aligarh 202002, India; haris6229@gmail.com (H.S.); owais_lakhnawi@yahoo.com (M.O.)

**Keywords:** graphite powder, graphene oxide, reduced graphene oxide, *Chenopodium album*, anticancer, antimicrobial

## Abstract

A novel method of preparing reduced graphene oxide (RGOX) from graphene oxide (GOX) was developed employing vegetable extract, *Chenopodium album*, as a reducing and stabilizing agent. *Chenopodium album* is a green leafy vegetable with a low shelf life, fresh leaves of this vegetable are encouraged to be used due to high water content. The previously modified ‘Hummers method’ has been in practice for the preparation of GOX by using precursor graphite powder. In this study, green synthesis of RGOX was functionally verified by employing FTIR and UV-visible spectroscopy, along with SEM and TEM. Our results demonstrated typical morphology of RGOX stacked in layers that appeared as silky, transparent, and rippled. The antibacterial activity was shown by analyzing minimal inhibitory concentration values, agar diffusion assay, fluorescence techniques. It showed enhanced antibacterial activity against Gram-positive and Gram-negative bacteria in comparison to GOX. It has also been shown that the synthesized compound exhibited enhanced antibiofilm activity as compared to its parent compound. The efficacy of RGOX and GOX has been demonstrated on a human breast cancer cell line, which suggested RGOX as a potential anticancer agent.

## 1. Introduction

Ever rising consciousness about human health, the addition of fruits and vegetables are gaining importance in their regular diet [1]. It has been reported that 39% of food waste occurs in the food production industry, and overall waste was approximately 126.2 million tons in 2020 [2]. Some parts of agricultural waste can effectively be utilized for the synthesis of graphene, and the same can also be used to obtain reduced graphene oxide (RGOX) from graphene oxide (GOX) [3]. 

*Chenopodium album* is also known as ‘bathua’, a leafy vegetable having a low shelf life due to its high moisture content [4]. It cannot be preserved for a longer duration, and it goes as a waste product, so liquid extraction of *Chenopodium album* can be stored and used as a reducing agent as well as a stabilizing agent. *Chenopodium album* is an excellent source of high-grade vitamins, proteins, nutrients and antioxidant predominantly retinol and ascorbic acid. The other health benefits of fiber rich *Chenopodium album* leaves include its laxative properties helpful in curing constipation, which in turn are useful in the treatment of piles. Furthermore, it improves the hemoglobin level, appetite, and purifies blood, which is beneficial for the human heart [5]. 

It was reported that graphene oxide nanosheets with enhanced peroxidize like activity used for electrochemical cancer cell detection and targeted therapeutics [6]. Furthermore, there were many reports which show the efficacy of graphene oxide-graphene quantum dots on cancerous cells [7]. The researchers used ascorbic acid, amino acids, reducing sugars, and protein bovine serum albumin as reducing as well as stabilizing agents [8]. The reducing chemicals are highly toxic, hazardous and may introduce impurities into RGOX, which is harmful to the environment and may impart adverse effects on its biological applications [9,10]. It was also reported that graphene oxide with silver nanocomposites exhibits excellent anticancer properties [11]. Guo and Mei reported that the graphene oxide with silver nanocomposites have bactericidal activity against *E. coli* through disrupting the integrity of the bacterial cell wall and showed a bacteriostatic effect on *S. aureus* cell division [12]. Another report demonstrated that GOX could be used in biomedical applications such as targeted drug delivery to the lung. Additionally, properties such as significant retention, better accumulation, remarkable pathological changes make GOX an efficient drug [13,14]. Contrary to the above facts, GOX is also reported to be involved in oxidative stress formation and induces cell cytotoxicity at high concentrations [15]. 

The formation of RGOX by different methods such as thermal [16], electrochemical [17], or chemical reductions process [18,19] has been reported in various research. In this study, graphite was oxidized to GOX, while conversion of graphene oxide (GOX) to RGOX was assigned to the reduction method with different strong reducing agents such as hydrazine (N_2_H_2_) [20] and sodium hydride (NaH) [21]. These reducing agents are highly toxic, hazardous, and pose a serious threat to our environment as well as an effect on biological activities. The assessment consequently attained an existent preparation of RGOX linked by preceding work conveyed designed for a diverse natural reducing agent such as lemon juice [22], tea leaves extract [23], fresh carrots [24], glycylglycine [25], *Euphorbia wallichi* leaf [26], *Abelmoschus esculentus* [27] and L-valine [28]. One of the studies showed that *Olax scandens* leaf extract bio-mediated conversion of silver and copper salts to a nanocomposite structure [29]. Similarly, the novel biological synthesis of RGOX was prepared by graphene oxide with plant extract *Chenopodium album* as a reducing agent. This biological synthesis of RGOX from GOX involving of *Chenopodium album* is cost-effective and less toxic as compared to synthesis by chemical reducing agents. The reduction parameters were investigated by UV-visible spectroscopy, FTIR, SEM and TEM. Moreover, antibacterial and anticancer activities of the synthesized RGOX and GOX activity have been evaluated. RGOX showed promising antibacterial and anticancer activities on breast cancer cell lines, ensuring a promising approach for its future treatment. 

## 2. Materials and Methods

### 2.1. Chemicals and Reagents

All the chemicals and reagents utilized in the experiment were AR (analytical reagent) grade. Natural graphite fine powder which was used was from the CDH company, concentrated sulfuric acid (H_2_SO_4_) and potassium permanganate (KMnO_4_) were from the BDH company, hydrogen peroxide (30% H_2_O_2_), and NaNO_3_ (Laboratory reagent) were from qualikems and concentrated HCl was from Loba Chemie Laboratory reagents and fine chemicals, New delhi, India. Phosphate buffer saline (PBS) (pH = 7.4), 3-(4, 5-dimethylthiazol-2-yl)-2, 5-diphenyl-tetrazoliumbromide) MTT dye, Roswell Park Memorial Institute (RPMI) media were purchased from Sigma-Aldrich and Invitrogen (New Delhi, India). Fluorescein isothiocyanate (FITC) dye for fluorescence microscopy was purchased from Sigma Aldrich. Bacterial culture media Agar, Brain Heart Infusion (BHI), and Luria Bertani (LB) were procured from Himedia Laboratories Pvt. Ltd. (New Delhi, India). The bacterial strains used were Gram-negative *Escherichia coli* (ATCC-25922), Gram-positive *Staphylococcus aureus* (ATCC-25923), and fungal strain *Candida albicans* (MTCC-183) was also used. All the chemicals were used as received without any further treatment. Deionized or doubly distilled water (DDW) was used throughout the experiments.

### 2.2. Preparation of Reduced Graphene Oxide (RGOX)

Initially, graphene oxide was prepared from graphite powder using the Hummers method in which 4 g of graphite were taken with sodium nitrate and concentrated sulfuric acid mixed in a round bottom flask is kept on a magnetic stirrer for 30 min [27,30]. After that, 12 g of potassium permanganate was added slowly while stirring the reaction mixture continuously under ice cooling with fast stirring keeping the temperature at 20 °C. At the end of 30 min, instead of the ice bath, the blends were kept inside an oven at 35 °C for 2 h followed by magnetic stirring. Afterward, 200 mL of deionized water was poured into the flask leading to the appearance of the brownish solution. At the end of 15 min, a mixture of 30% of hydrogen peroxide and toasty DMW was included continuously until the brownish color solution changed into a yellowish-brown color solution. Finally, the product of the solution was filtered by Whatman filter paper and washed with hydrochloric acid and double-distilled water. The obtained brown powdered graphite oxide was kept dry in an oven at 50 °C overnight and then the yielded graphene oxide (100 mg) was suspended in 100 mL of double-distilled water using an ultrasonicator. The supernatant of graphene oxide was obtained by centrifugation and then stored for experimental use. While the synthesis of reduced graphene oxide (RGOX) used a seasonal plant fresh *Chenopodium album.* Firstly, 500 g of vegetable was added into the distilled water followed by boiling for about 15 min until a green color was obtained and vegetable waste was eliminated using Whatman filter paper. Eventually, the suspension of 500 mg graphene oxide in 500 mL DDW was added into the extract of *Chenopodium album*, then the blend was kept on reflux for 12 h at 100 °C for reduction. After filtration, bio-mediated reduction of graphene oxide turned from a brownish solution into a black colored solution, which was further heated to a fine powder of RGOX. It was then washed with DMW thoroughly and thus finally filtered to obtain reduced graphene oxide. Furthermore, the synthesized reduce graphene oxide was further characterized by different techniques.

### 2.3. Characterization of Graphene Oxide (GOX) and Reduced Graphene Oxide (RGOX)

Four characterization techniques were used for the confirmation of GOX and RGOX. UV-visible spectroscopy (Hitachi-F-2500-, Tokyo, Japan). and Fourier Transforms Infrared Spectroscopy (FTIR) (PerkinElmer, Spectrum-BX, Norwalk, CT, USA). were used for the confirmation of the formation of GOX and RGOX. Superficial framework and magnitude of GOX and RGOX were confirmed by Scanning electron microscopy (SEM) and Transmission Electron Microscopy (TEM), respectively. The absorbance range of the FTIR spectroscopy technique was 400 to 4000 cm^−1^. SEM images were taken using a (JSM-6510LV, Jeol, Tokyo, Japan) while an advanced JEOL 6510LV model was used to obtain higher resolution images. The SEM was operated with a working energy of 7 keV, a beam size at a value of 3, and a working distance of 10 mm. TEM analysis was performed employing a (JEM 2100, JEOL, Tokyo, Japan) model with a potential of 200 kV FE (Field Emission). Powder X-ray diffraction (XRD) patterns were characterized using XRD on a Bruker D8 Advance with Cu Kα radiation in a scanning range of 5–60 (2 theta) with a scan rate of 12°/min.

### 2.4. Determination of Minimum Inhibitory Concentration (MIC)

The minimum inhibitory concentration (MIC) value of in situ synthesized RGOX was calculated by the micro-dilution method. MIC is the minimum concentration of synthesized compounds that inhibit the visible growth of microorganisms after a given time. Bacterial and fungal strains such as *Staphylococcus aureus* (ATCC 25923), *Escherichia coli* (ATCC-25922), and *Candida albicans* (MTCC-183) were tested against synthesized compounds GOX and RGOX. The significant values were obtained on the basis of the visibility test conducted in 96-well microdilution plates, as described previously [31]. Positive controls were prepared without compounds and only cells, while negative control was prepared without compound and cells.

### 2.5. Antimicrobial Activity Determination by Agar Well Diffusion Assay

The antimicrobial activity of GOX and RGOX was determined by agar well diffusion assay against *Staphylococcus aureus, Escherichia coli,* and *Candida albicans*. An aliquot of 100 µL of cells was taken from a stock of 10^5^ CFU/mL of different microbial strains that were spread on agar plates and incubated at 37 °C for a stipulated time period. The wells were punched into the agar plates with the help of sterile yellow tips, and 50 µL of synthesized compounds were loaded and incubated overnight at 37 °C. The zone of inhibition of GOX and RGOX and standard drugs (like ampicillin for bacteria and amphotericin B for fungus) was measured after overnight incubation. The readings were taken in triplicates to reduce deviations, and calculation should be done after taking an average of triplicates.

% activity index = zone of inhibition by test compound (diameter)/zone of inhibition on by standard (diameter) × 100%

### 2.6. Biofilm Inhibition Employing Fluorescence Microscopy

*Staphylococcus aureus* (ATCC-25923) was cultured overnight in Brain Heart Infusion (BHI) broth. It was then dispended (10^6^ CFU/mL) on a sterile coverslip in a six-well culture plate (Eppendorf India Limited). Then the cells were incubated for 24 h at 37 °C for the growth of biofilm. After incubation, washing with PBS was done to remove non-adherent cells and then treated with GOX and RGOX. After a stipulated time period, the plates were washed with PBS, and coverslips were fixed with 4% paraformaldehyde followed by washing and staining with FITC dye. After 30 min, stained coverslips were washed with PBS and analyzed under a fluorescence microscope (100× magnification) [32].

### 2.7. XTT Biofilm Assay

2,3-bis(2-methoxy-4-nitro-sulfophenyl)-5-[(phenylamino)carbonyl]-2H-tetrazolium hydroxide (XTT) biofilm assay was performed to analyze the behavior of GOX and RGOX according to a previously published protocol [33]. Briefly, mature biofilms were grown on cover slides, and non-adherent cells were washed thoroughly with sterile phosphate saline buffer (PBS). Then mature biofilm was exposed to increasing concentrations of GOX and RGOX incubated for 48 h. After incubation, XTT solution in PBS was added. Previously prepared 2 μL menadione solutions (0.4 mM) were added in each well and incubated at 37 °C in the dark for 4 h. The colorimetric variation was assessed by a micro titer plate reader (BIO-RAD) at 490 nm. 

### 2.8. Cell Cytotoxicity Assay

3-(4,5-dimethylthiazol-2-y1)-2,5-diphenyltetrazolium bromide (MTT) assay was performed on MCF-7 to determine the effect of GOX and RGOX compounds. Cells were cultured in Roswell Park Memorial Institute (RPMI) 1640 medium supplemented with 10% heat-inactivated fetal calf serum, 10 mmol/L glutamine, and 50 μg/mL each of streptomycin and penicillin. The cells were seeded in 96 well plates with a density of 1 × 10^5^ cells and incubated at 37 °C. GOX and RGOX solutions were freshly prepared for the exposure to cells, and increasing concentration of both compounds (0–250 µM) was added to the cells. After overnight incubation, the cells were washed with PBS, and 0.5% MTT solution (5 mg/mL in PBS) was added in each well. After 4 h incubation, formazan crystals were formed, which gives purple color by adding 0.1% DMSO solution. This colored complex was measured by Microplate Reader (Genetix Biotech Asia Pvt. Ltd., New Delhi, India) at 570 nm. Cisplatin was taken as a control in the experiment.

## 3. Results and Discussion

### 3.1. Ultraviolet-Visible Spectroscopy (UV-Vis) Analysis

**Figure 1:**
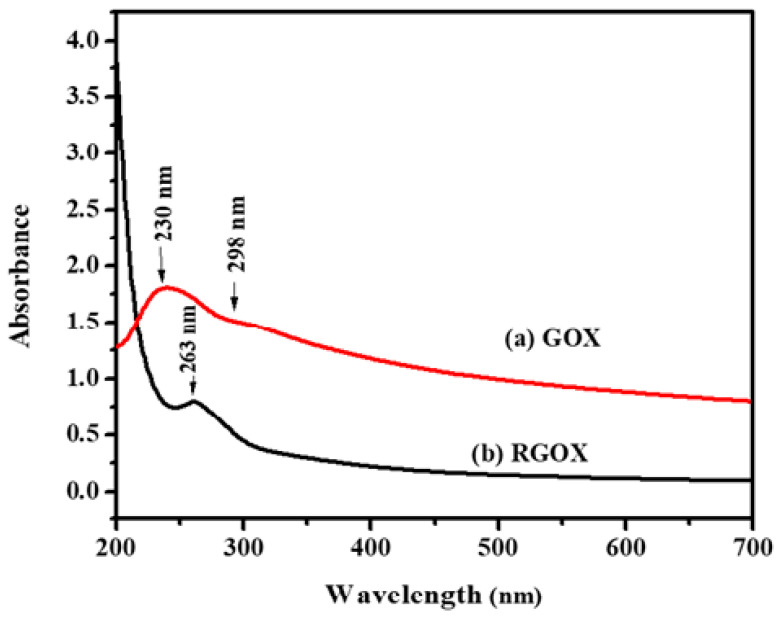
Ultraviolet-visible spectra analysis (**a**) GOX and (**b**) RGOX.

The confirmation of synthesized GOX and then reduction of GOX into RGOX by extract vegetables were investigated through UV visible spectroscopy, as shown in Figure 1a,b. The absorbance peak of GOX due to the π→π* transition of aromatic C=C at 230 nm, However the hump of GOX alongside 298 nm was observed and assigned as the nonbonding between the π antibonding transition of the carbonyl group [27,34]. Eventually, the reduction of GOX formed RGOX that shows the absorbance peak at 263 nm, while the hump of GOX disappeared. Finally, complete removal of oxygen functional groups in the GOX confirmed the formation of RGOX, and the resultant reduce graphene oxide by the network of π-conjugation of graphite [35]. One more superior distinguishing feature of GOX and RGOX, the appearance of a shoulder peak around 230 nm due to the transition of aromatic compound C-C bonds which resemble sp^3^ hybridization, on the other hand after conversion of GOX into RGOX by reduction of vegetable extract which observed a clear peak at 263 nm through sp^2^ hybridization. Extract of the *Chenopodium album* consists of many antioxidants such as vitamin A, vitamin C, vitamin B, flavonoids, etc. In this work, the conversion of graphene oxide into reduces graphene oxide was probably achieved due to the reducing nature of vitamin C and other antioxidants present in the extract of the *Chenopodium album*. Some of the studies have also highlighted the green, reducing nature of these antioxidants [36,37].

### 3.2. Fourier Transforms Infrared Spectroscopy (FTIR) Analysis

**Figure 2:**
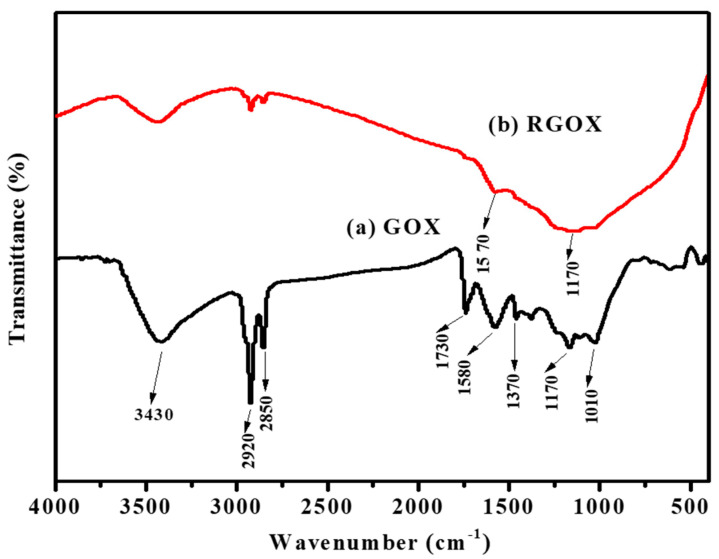
Fourier transforms infrared analysis (**a**) GOX and (**b**) RGOX.

FTIR spectroscopy was used to verify the synthesis of graphene oxide and material RGOX. FTIR spectrum confirmed of GOX was obtained by complete oxidation of graphite and also confirmed RGOX, as shown in Figure 2. The GOX spectrum displays a broad peak at 3430 cm^−1^ [38], while a number of strong band absorptions at 1010, 1170, 1370, 1580, 1730, 2850 and 2920 cm^−1^, were observed. The broad absorption peak and 1370 cm^−1^ are attributed to the C-H group, while the peak about 1010 cm^−1^ corresponds to the C–O–C bond of the alkoxy or epoxy group. The IR peak is attributed to 2920, 2850, and 1580 cm^−1^ due to the asymmetric CH_2_, symmetric CH_2_ stretching of GOX, and C=C bond stretches without an oxidized graphitic domain respectively [11,39]. The peak around at 1170 cm^−1^ is due to C–OH bonds, and 1730 cm^−1^ is associated with the C=O stretch of carbonyl group [40]. In GOX, the functional group-containing oxygen was probably reduced due to the presence of antioxidants such as vitamin C, ascorbic acid present in *Chenopodium album* as shown in Figure 2. The FTIR peak of RGOX at 1570 cm^−1^ is related to elongating of C=C, besides the broad peak at 1170 cm^−1^, which is assigned to the C–C stretching. The spectra of RGOX as compared to GOX, the band at 1730 cm^−1^ (associated with C=O in carbonyl group), and 1010 cm^−1^ (associated with C–O–C in the epoxy group) disappeared; this indication confirmed that GOX was reduced to RGOX. Eventually, some strong peaks associated with oxygen-containing functional groups decreased for reduced graphene oxide as compared to graphene oxide.

### 3.3. X-Ray Diffraction (XRD) Analysis.

**Figure 3:**
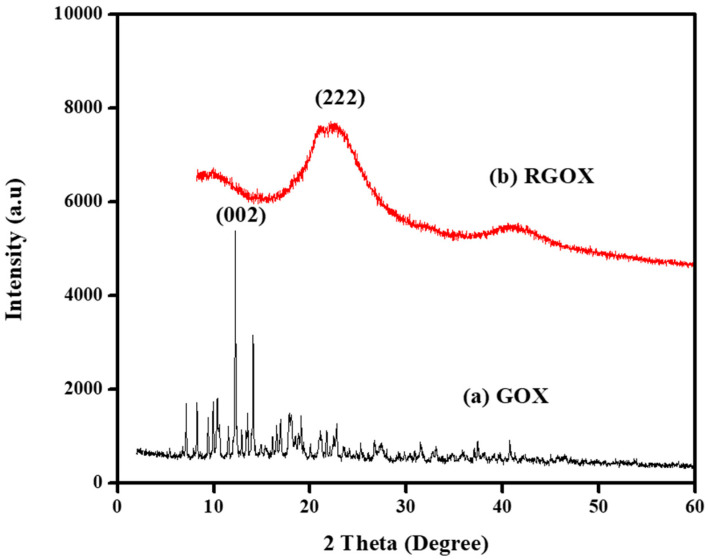
XRD graph of (**a**) GOX and (**b**) RGOX.

XRD configuration of GOX and RGOX is depicted in Figure 3a,b. The spectra observed for GOX and RGOX were crystalline and amorphous in nature. However, GOX shows, with respect to its characteristics, a band at 2θ = 12.50° (002) as shown in Figure 3a, after oxidation of graphite that suggests water molecules were inserted into the crystal lattice layer and different functional groups of oxygen were produced between the graphite layers [41]. Thus, after the reduction of GO from vitamin C, most oxygen functional groups (C–OH, C–O and C=O) were removed where a sharp peak at 2θ = 12.50° was extinct. In addition, XRD of RGOX, a diffused peak band appeared at 2θ = 22.50° and corresponded to diffraction (222), as depicted in Figure 3b [42].

### 3.4. Scanning Electron Microscopy (SEM) and Transmission Electron Microscopy (TEM) Analysis

**Figure 4:**
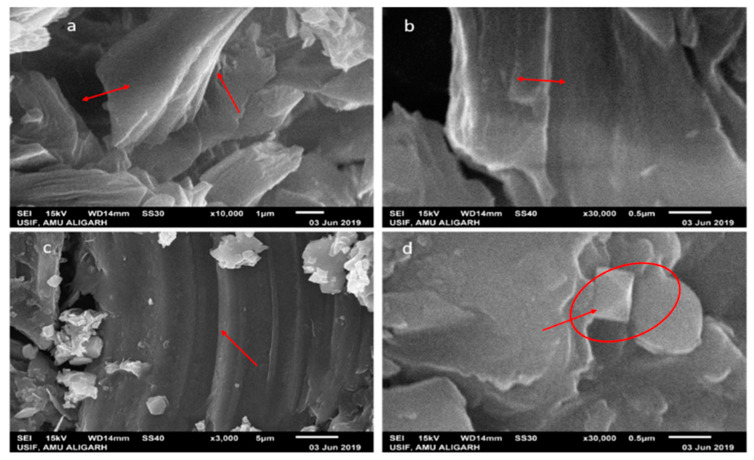
Scanning electron microscopy images at different magnifications (**a**) GOX at 10 K, (**b**) GOX at 30 K, (**c**) RGOX at 3 K and (**d**) RGOX at 30 K.

**Figure 5:**
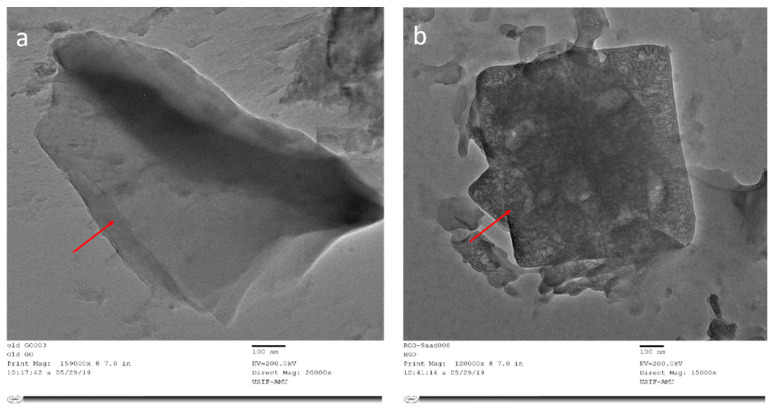
Transmission electron microscopy images of (**a**) GOX and (**b**) RGOX.

The morphology of the scanning electron micrograph shows the two types of GOX sheets, independent flat GOX mass with crumples and intact as shown in Figure 4. In Figure 4a at 10,000 times magnification, you can see folding and overlapping and there is a large interspace between the thinner edges of graphene oxide. It is clear from the micrograph that the differential thermal factors amongst graphene sheets and the substrate shrunk throughout the practice formed by annealing [43]. While in Figure 4b at 30,000 times magnification, the GOX sheets overlapped as same, but they were slippery, and there was no interspace between the thinner edges of graphene oxide. Additional SEM examination indicates that the full information on distinct RGOX flecks of layers and edge layers had a distribution of shady graphene flecks. At 3000 times magnification, the entire sheet of RGOX was visible exposed as assigned in Figure 4c. However, at 30,000 times magnification, RGOX overlaid descriptive characteristics of graphene structures and crimped fragments were well scattered and linked with each other as shown in Figure 4d. The number of films can be seen by the distinct contrast obtained in the SEM images. TEM morphology of graphene oxide and reduce graphene oxide are depicted in Figure 5. The surface study of graphene oxide manifested a single layer, without wrinkles, with an irregular pattern meant an amorphous nature and flaw structure. Thus, in the TEM images of RGOX many more wrinkle shapes can be observed with multiple layers stacked on each other. It is signified that the scrolled shape and lateral corrugations investigated by TEM [44].

### 3.5. Minimal Inhibitory Concentration of GOX and RGOX against Bacterial and Fungal Isolates

MIC values were determined by the microdilution method in 96-well plates on the basis of a viability test, as previously described [29,45]. Both bacterial and fungal cultures were adjusted to 1 × 10^5^ colony-forming unit (CFU)/mL with their respective media. The stock sample of the formulations was then serially diluted in the 96-well plate. Furthermore, both positive and negative controls were taken without compound, and without compound and organisms, respectively. The plate was then incubated overnight at 37 °C. On the next day, the turbidity of the cells in wells was checked as an indicator of microbial growth. Therefore, the well which showed no growth of microbes was the minimum inhibitory concentration of RGOX and GOX.

The antibacterial potential of GOX and RGOX nanocomposite was determined against Gram-positive and Gram-negative bacteria. Ampicillin has been taken as a control antibiotic. The MIC value of GOX against *Staphylococcus aureus* and *Escherichia coli* was 250 μg/mL, whereas RGOX had a MIC value of 125 μg/mL against both strains. The control ampicillin had MIC values of 4 and 2 μg/mL for *Staphylococcus aureus* and *Escherichia coli*, respectively. Similarly, the MIC values of GOX and RGOX were calculated against *Candida albicans* and found to be 500 μg/mL and 500 μg/mL, respectively, in which amphotericin B was taken as a control.

### 3.6. Agar Diffusion Assay

The antibacterial efficacy of GOX and RGOX was determined by agar diffusion assay. The formation of a zone of inhibition by these compounds suggested the bactericidal activity of nanocomposites against different bacterial strains. The bactericidal effect was conducted against *E. coli*, *S. aureus*, and *C. albicans* on a nutrient agar plate containing different concentrations of various formulations. It was observed that only the bacterial strains were sensitive to RGOX and GOX while fungal strain was insensitive towards these compounds. The zones of inhibition recorded against these are reported in Table 1. The zones of inhibition of *S. aureus* and *E. coli* increased in RGOX as compared to GOX, while both compounds did not show any effect on *C. albicans*. Furthermore, our results were in concordance with Shen et al., who investigated the antibacterial activity of an Ag-CCG composite against *Colibacillus*, *Staphylococcus aureus* and *Canidia albicans* bacteria [46]. As suggested by the results, *Chenopodium album* synthesized RGOX with considerably better antibacterial activity compared to its parent compound GOX. RGOX and GOX did not show antifungal activity on fungus *Candida albicans*.

### 3.7. Antibiofilm Activity Revealed by Fluorescence Microscopy by GOX and RGOX

**Figure 6:**
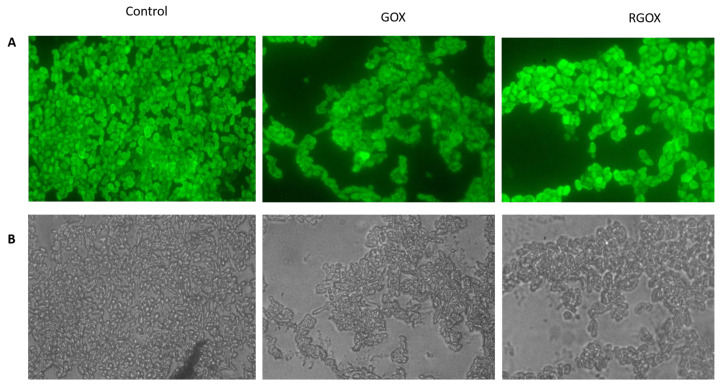
Inhibition of *S. aureus* biofilm by synthesized compounds: (**A**) shows fluorescence microscopic images while (**B**) shows bright-field microscopic images, the three panel shows untreated control image of *S. aureus* biofilm, biofilm treated with GOX, biofilm exposed to RGOX as revealed by fluorescence microscopy (100× magnification).

GOX and RGOX showed inhibition of biofilm formation, Figure 6. As compared to the control, in which no compound was used, the results of GOX and RGOX showed enhanced antibiofilm potential by successfully disrupting *Staphylococcus aureus* biofilm. The FITC labeled fluorescence images (100×) showed the disruptions of *Staphylococcus aureus* biofilm in RGOX and GOX; the panel revealed the antibiofilm activity.

### 3.8. XTT Assay Employing to Determine Anti-Biofilm Potential of GOX and RGOX

**Figure 7:**
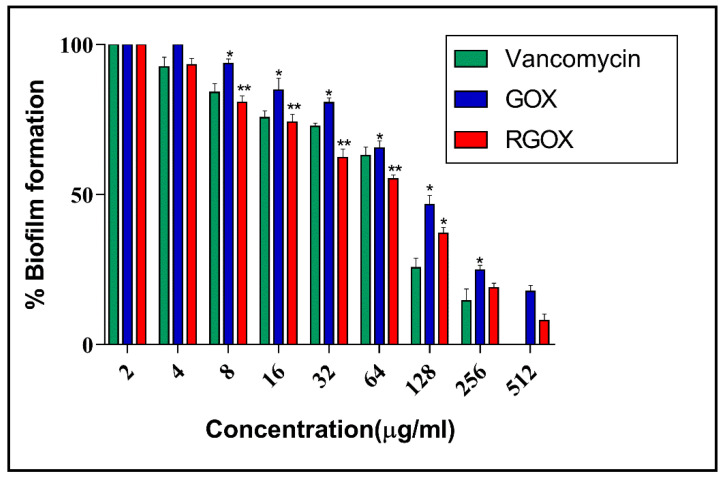
GOX and RGOX effect against *S. aureus* biofilm development. An increasing concentration of compounds decreases the percentage of biofilm formation. Vancomycin served as a control (*p*-value < 0.05 *, < 0.01 ** and < 0.001 ***).

The antibiofilm nature of GOX and synthesized RGOX has been investigated against *S. aureus* with vancomycin antibiotic as a control. The formation of biofilm depends on numerous factors, such as extracellular binding proteins, polysaccharides, etc. The inhibitory action of RGOX on biofilm is caused due to the internal metabolic system, which disrupts the formation of extracellular protein factors responsible for the formation of biofilm. In the present study, *S. aureus* was cultured in a 96-well plate and treated with varying doses of compounds. The dose-dependent effect of GOX and RGOX was found to exhibit antibiofilm activity by employing XTT assay on an *S. aureus* strain. A significant decrease in the bacterial numbers (*p*-value < 0.05 * and < 0.01 **) was found in both treated groups as compared to vancomycin treatment. This observation is in agreement with the zone of inhibition assay. As the dose of the compound increased, the percentage of biofilm decreased, as shown in Figure 7. Growth inhibition was calculated by comparing the relative metabolic activity (RMA) obtained by taking the untreated control as 100% by XTT.

### 3.9. Cytotoxicity of GOX and RGOX Towards MCF-7 Cells

In this study, we evaluated the cytotoxicity of RGOX towards MCF-7 (human breast cancer cell line) by employing an MTT assay. The fluorinated graphene oxide showed no cytotoxicity towards breast cancer cells at a concentration of 576 µg/mL [47] Moreover, graphene oxide showed cytotoxicity in HBI.F3 human neuronic cells and BEAS-2B human lung cells at a dose-dependent concentration of 10–100 μg/mL [48]. In most of the previous studies, it was seen that graphene oxide shows a cytotoxic effect on breast carcinoma at a very high concentration above 500 μg/mL.

**Figure 8:**
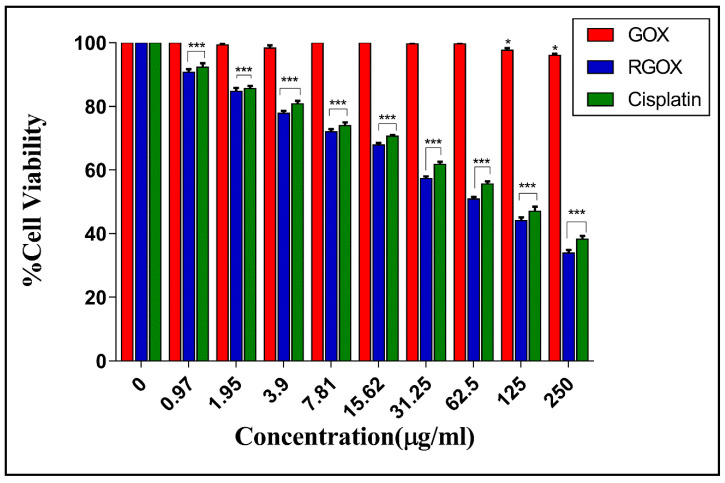
Cytotoxicity assay of MCF-7 cancer cells with increasing concentrations (0–250 μg/mL) of Cisplatin, GOX, and RGOX (p-value < 0.05 *, < 0.01 ** and < 0.001 ***).

According to Liao et al., the cytotoxicity of skin fibroblasts enhanced with the increase in the concentration of graphene oxide [49]. In addition to that, an increasing concentration of GOX amplified the cytotoxic effect on HepG2 cells [50]. In one of the studies, it was observed that the synergistic effects of RGOX and ZnO nanorods produced excellent cytotoxic effects in human embryonic kidney cells (HEK293) with the help of enhanced antioxidant properties. This is because zinc ions attached to RGOX sheets contacted with HEK293 cells and cause dthe disruption of cells [51]. In one of the dose-dependent studies, an increasing dose of GO-FA-ZnO from 0 to 100 μg/mL reduced cell viability up to 19% as compared to a control [52]. Previously, it was shown that the GO-Ag toxicity towards breast cancer cells may be synergistic. Breast cancer cells were treated with different concentrations of GO-Ag nanocomposite in a dose-dependent manner (10–100 µg/mL), which decreased cell viability [53]. As compared to earlier studies, the bio-mimetically synthesized RGOX was used to determine its effect on the viability of breast cancer cells. In a dose-dependent study, it was found that the *Chenopodium album* synthesized RGOX showed decreased cell viability from 90% to 35% towards MCF-7 cells at a concentration from 1 to 250 µg/mL. Furthermore, the *Chenopodium album* synthesized RGOX compound was found to be more cytotoxic as compared to the control GOX. Figure 8 shows clearly the loss of cell viability when the concentration increased gradually (in a dose-dependent manner). Therefore, the toxicity of bio-mimetically synthesized RGOX towards breast cancer cells may be synergistic, which disrupted the interaction between cancerous cells.

## 4. Conclusions

Graphene oxide (GOX) was successfully prepared using graphite powder by a modified ‘Hummers method’. GOX was satisfactorily reduced to RGOX in the presence of *Chenopodium album* leaves extract. The present study is the first to report this green, simple, facile and cost-effective procedure for direct reduction of GOX using *Chenopodium album* leaves extract. The obtained GOX and RGOX were characterized using UV and FTIR techniques, which show the negligible presence of oxygen functional groups in GOX during the reduction process. *Chenopodium album* is a good source of vitamin C, casein, caffeic acid, and polyphenols, which together provide reducing ambiance. UV, FTIR, SEM, and TEM analysis confirmed the formation of RGOX from graphene oxide using *Chenopodium album* leaves extract. The overall results of the experiments prove that *C. album* leaves extract is an important alternative to the traditional chemical reduction method to avoid chemical intervention. The activity of GOX and RGOX against bacteria and fungi has been demonstrated. It was found that RGOX showed an increased antibacterial (Gram-positive and Gram-negative) and antibiofilm activity as compared to GOX. The antifungal activity could not be ascertained using the synthesized compound. Upon evaluation of the anticancer activity of RGOX, it was found that it showed better anti-breast cancer activity than its parent compound GOX. The results of RGOX against MCF-7 cells will pave the way for a new approach for the prevention of breast cancer. RGOX obtained in this work can be further utilized in several potential applications in various fields such as drug delivery, anticancer, antifungal, antibacterial (Gram-positive and Gram-negative), DNA binding interaction, generation of bio-composites and biosensors. 

## Figures and Tables

**Table 1:** Zones of inhibition observed (in mm units) against microbial strains.

**Bacterial Strains**	**GOX**	**RGOX**
*S. aureus*	6.6 ± 2.0	8.6 ± 3.2
*E. coli*	6.3 ± 2	7.6 ± 2.0
**Fungal strain**	**GOX**	**RGOX**
*C. albicans*	1.4 ± 2.4	2.3 ± 2.09
